# Pervaporation separation of ethyl acetate from aqueous solutions using ZSM-5 filled dual-layer poly(ether-*block*-amide)/polyethersulfone membrane

**DOI:** 10.1039/c7ra13382k

**Published:** 2018-01-26

**Authors:** M. Vatani, A. Raisi, G. Pazuki

**Affiliations:** Department of Chemical Engineering, Amirkabir University of Technology (Tehran Polytechnic) Hafez Ave, P. O. Box 15875-4413 Tehran Iran raisia@aut.ac.ir +98 21 66405847 +98 21 64543125

## Abstract

In the present study, dual-layer mixed matrix membranes (MMMs) were prepared by incorporating ZSM-5 zeolite into poly(ether-*block*-amide) (PEBA) as an active layer on the polyethersulfone (PES) membrane as a support layer for pervaporation separation of ethyl acetate (EAc) from EAc/water mixtures. The ZSM-5 zeolite nanoparticles were synthesized by the hydrothermal technique and characterized using XRD, XRF and FESEM analysis. The ATR-FTIR, SEM, DSC and contact angle tests were used to characterize the fabricated MMMs. The effect of ZSM-5 concentration on the performance of the membranes was investigated by the pervaporation experiments and the results showed that loading 10% wt% ZSM-5 into the PEBA matrix had the best separation performance. The effect of feed concentration (1–5 wt%) and operating temperature (30–50 °C) on the separation factor and permeation flux of the neat PEBA/PES and PEBA/PES membranes containing 10 wt% ZSM-5 were studied at laminar and turbulent feed flow regimes. Analysis of variance was used to investigate the interaction effect of EAc concentration and temperature on the performance of the prepared membranes. It was observed that both feed concentration and temperature had positive effects on the total permeation flux and separation factor. The ZSM-5/PEBA/PES membrane containing 10 wt% ZSM-5 showed a separation factor and total flux of 124.94 and 1882 g m^−2^ h^−1^ at laminar flow and 134.22 and 1985 g m^−2^ h^−1^ at turbulent flow, respectively for a feed concentration of 5 wt% and temperature of 50 °C.

## Abstract 2

Dual-layer mixed matrix membranes were prepared by incorporating ZSM-5 zeolite into PEBA as an active layer on the PES membrane as a support layer for pervaporation separation of EAc from the EAc/water mixtures.

## Graphical abstract

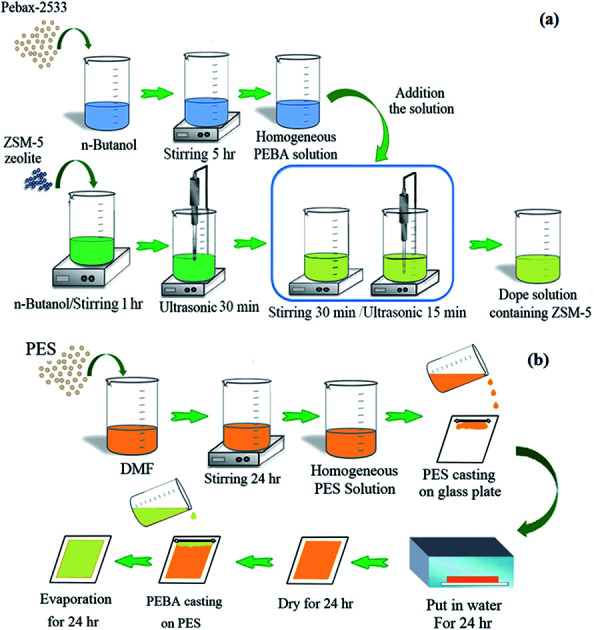

## Introduction

Pervaporation, as a membrane separation technology, is an efficient method for the separation of organic compounds from aqueous solutions in comparison to conventional processes like distillation due to energy-saving process, low production costs and high selectivity.^1^ Three types of materials including polymers, inorganic and composite materials have been used for fabrication of the pervaporation membranes.^2^ The hydrophobic polymers were used to enhance the efficiency of polymeric membranes toward the separation of organic compounds with low concentrations from aqueous solutions.^3–5^ However, the use of polymeric membranes due to the low chemical and thermal stability as well as intrinsic trade-off between permeability and selectivity is limited.^6^ The higher chemical and thermal stability were obtained by preparing inorganic membranes.^7^ However, the main drawback of inorganic membranes is the high brittleness and difficult fabrication of inorganic defect-free membranes in the large scale.^8^ Mixed matrix membranes (MMMs) have been prepared from the polymeric and inorganic materials to overcome the drawbacks of neat polymeric and inorganic membranes.^9^ The MMMs were prepared *via* different methods such as physical blending of the polymer and inorganic materials,^10^ sol–gel method,^11^*in situ* polymerization^12^ and self-assembly method.^13^ Among them, the physical blending of polymer/inorganic materials and sol–gel methods were commonly used for fabrication of the pervaporation MMMs. However, in the development of the MMMs, there are several challenges such as inorganic particles agglomeration and sedimentation, interfacial voids, low adhesion, polymer chains rigidification and pore blockage which have significant effects on the separation performance of the pervaporation membranes.

Various inorganic materials as filler including activated carbon,^14^ graphene oxide,^15^ carbon nanotubes,^16^ metal oxide nanoparticles,^17^ clay,^18^ mesoporous materials^19^ and zeolites^20^ have been used for the preparation of MMMs. Among them, zeolites with crystalline structures and well-defined pores in the range of nanometers due to their good adsorption properties as well as high mechanical and chemical stability have been widely employed for the preparation of pervaporation MMMs. Different zeolites such as NaA,^21^ NaY,^22^ NaX,^23,24^ ZIF-8 (ref. 25) and ZSM-5 (ref. 26–30) have been blended with polymers for the fabrication MMMs in the pervaporation process. Due to its high hydrophobicity, high surface area and uniform pore size distribution, ZSM-5 zeolite has been incorporated in the polymeric membranes to develop hydrophobic pervaporation MMMs toward the separation of organic compounds from aqueous solutions. For example, Zhang *et al.*^26^ prepared the ZSM-5/hydroxyl terminated polybutadiene (HTPB)-based polyurethaneurea (PU) MMM for the pervaporation separation of isopropyl acetate from its aqueous solutions. Kittur *et al.*^28^ fabricated the ZSM-5/PDMS MMMs for the pervaporation separation of isopropanol from water. Vane *et al.*^29^ also incorporated the ZSM-5 zeolite into the PDMS membranes for the separation of ethanol from ethanol/water mixtures. Gu *et al.*^30^ fabricated the single layer ZSM-5/poly(ether-*block*-amide) (PEBA) membrane for the separation of ethyl acetate (EAc) from aqueous solutions.

Multi-layer mixed matrix membranes are another type of the membranes that have been used in the pervaporation process in recent years. The multi-layer mixed matrix membranes consist of a thin dense top layer containing inorganic fillers on porous sub-layers as a mechanical support. In previous studies, we reported the performance of nano zeolite NaX/polyvinyl alcohol (PVA)/polyethersulfone (PES)^31^ and carbon nanotubes/PVA/PES/polyester^32^ multilayer mixed matrix membranes for dehydration of ethanol/water mixture *via* the pervaporation process. However, there are few studies about pervaporation separation of organic compounds from aqueous solutions using multi-layer pervaporation MMMs.

Due to its low cost and low toxicity, EAc is widely used as a solvent in the chemical industry and as a raw material for the production of plasticizers, adhesive agents, drugs, perfumes, thinners, synthetic resins and varnishes.^33,34^ EAc is mainly synthesized *via* the esterification of acetic acid with ethanol.^35^ The product of the esterification process consists of water. Therefore, the EAc produced from the esterification process has a low concentration and needs to be concentrated and purified for use in various applications. In this area, the pervaporation, as an energy saving membrane process, has a high potential for the recovery of EAc from the aqueous solutions.^36^ In this work, the ZSM-5/PEBA/PES dual-layer MMMs were prepared by casting ZSM-5/PEBA/*n*-butanol solution over the porous PES membrane as a support layer. The ZSM-5 zeolite nanoparticles were synthesized *via* the hydrothermal method and characterized using XRD, XRF and FESEM analysis. The ATR-FTIR, SEM, DSC and contact angle analysis were used to characterize the prepared membranes. The effect of ZSM-5 content on the performance of prepared MMMs toward the recovery of EAc from aqueous solutions was investigated. Also, the effect of feed concentration and operating temperature on the separation factor and permeation flux of the dual-layer MMMs in the laminar and turbulent flow regimes was studied. A full factorial design was used to develop the polynomial models for the separation factor and total flux as a function of EAc concentration and temperature. The main innovative aspect of this study is the use of nanofillers to prepare dual-layer mixed matrix membranes for the pervaporation separation of the EAc from the aqueous solutions. Another important contribution is the investigation of the effect of feed concentration, temperature and flow rate on the separation performance of the prepared mixed matrix membranes with the analysis of variance (ANOVA).

## Experimental

### Materials

The commercial Pebax® 2533 (80 wt% poly(tetramethylene oxide) and 20 wt% polyamide) and PES (molecular weight of 58 000 g mol^−1^, Ultrason E6020P) provided from Arkema Inc. (Paris, France) and BASF (Ludwigshafen, Germany) were used as the membrane materials, respectively. *N*,*N*-Dimethyl formamide (DMF) and *n*-butanol purchased from Merck Co. Ltd. (Darmstadt, Germany) were used as solvents for PES and PEBA, respectively. Tetrapropylammonium hydroxide (TPAOH), sodium hydroxide (NAOH), aluminum isopropoxide supplied from Merck Co. Ltd. and tetraethyl orthosilicate (TEOS) supplied from Sigma Inc. (Sigma Aldrich, MO, USA) were used for synthesis of the ZSM-5 zeolite. Ethyl acetate was purchased from Merck Co. Ltd. and de-ionized water was mixed as the pervaporation feed.

### Synthesis of ZSM-5 zeolite

ZSM-5 zeolite nanoparticles were synthesized from gel composition containing TPAOH, TEOS and water as solution A and aluminum isopropoxide, NAOH, TPAOH and water as solution B by the following procedure. 4.22 g TPAOH and 8 g TEOS was initially dissolved in 20 g de-ionized water under stirring for 2 h (solution A). Solution B was prepared by dissolving 1.8 g TPAOH, 0.48 g NAOH and 0.3 aluminum isopropoxide in 20 g de-ionized water. Solution A and solution B were then mixed together under stirring for a further 4 h to obtain a homogeneous gel. The prepared gel was then put to a Teflon lined steel autoclave at 200 °C for 2 days. Afterward, the synthesized zeolite was centrifuged and washed with deionized water three times. Then, the obtained solid product was dried at 110 °C for 12 h. Finally, the synthesized zeolite was calcined at 550 °C for 5 h.

### Fabrication of dual-layer mixed matrix membranes

The phase inversion technique was used to fabricate the ZSM-5 filled PEBA/PES dual-layer MMMs. The porous PES ultrafiltration membrane was prepared as a support layer using the non-solvent induced phase separation (NIPS) method as previously described.^37–39^ Briefly, the air bubble-free 16 wt% PES solution is prepared by dissolving the PES granules into the DMF under stirring for 24 h followed by de-aerating in a vacuum system for 1 h. The PES membranes were prepared by casting the PES solution on a glass plate followed by immersing into the de-ionized water batch at room temperature, storing into another de-ionized water bath for 24 h and drying at room temperature for another 24 h. The thickness of the prepared membranes was about 80 ± 5 μm.

The neat PEBA and ZSM-5/PEBA membranes were prepared as a selective top layer on the surface of the PES sub-layer by the solvent evaporation induced phase separation method.^40^ Briefly, the PEBA homogenous solution was achieved by dissolving PEBA granules in *n*-butanol under stirring for 5 h at 70 °C. In addition, the suspensions of ZSM-5 zeolite were prepared by adding various amounts of ZSM-5 nanoparticles to *n*-butanol under stirring for 1 h and sonication for 30 min in an ultrasonic (UP200S (200 W, 24 kHz), Hielscher Ultrasonics GmbH, Germany). Then one sixth of the PEBA solution was added to the ZSM-5 suspension under stirring for 30 min followed by sonication in an ultrasonic for 15 min. This procedure was continued until all of the polymer solution was added to the zeolite suspension. Afterwards, the prepared bubble-free dope solution containing ZSM-5 was casted over the porous PES support membrane. Finally, the prepared films were dried at room temperature for 24 h, and then the residual solvent was evaporated by drying at 60 °C in an oven. The thickness of the top layer was about 20 ± 2 μm.

**Fig. 1:**
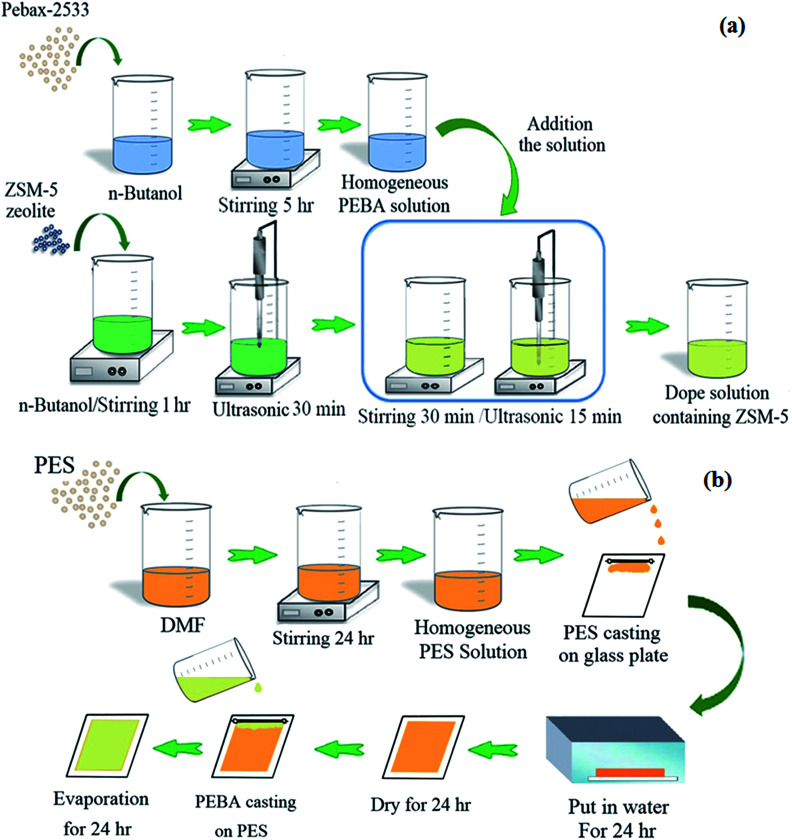
The schematic of procedure used for the preparation dope solution containing ZSM-5 nanoparticles (a) and fabrication of dual layer ZSM-5/PEBA/PES membrane (b).

Fig. 1 shows the schematic of the procedure used for the preparation dope solution containing ZSM-5 nanoparticles and fabrication of the PES membrane and dual-layer ZSM-5/PEBA/PES membrane. The fabricated membranes were named as given in Table 1.

**Table 1:** The nomenclature of various prepared membranes

Membrane sample	PEBA-2533 (wt%)	ZSM-5 (wt%)	*n*-Butanol (wt%)
M0	10	0.00	90.00
M05.0	10	0.50	89.50
M07.5	10	0.75	89.25
M10.0	10	1.00	89.00
M12.5	10	1.25	88.75
M17.5	10	1.75	88.25

### Characterization tests

#### XRD analysis

The powder's X-ray diffraction (XRD) instrument (Equinox 3000, Inel, France) at a temperature of 25 °C and a scanning speed of 0.03° (2*θ*) s^−1^ was used to confirm the structure of the synthesized ZSM-5 zeolite.

#### XRF analysis

The elemental composition of the synthesized ZSM-5 zeolite was determined using X-ray fluorescence (XRF) analysis by a Philips spectrometer (PW 1404 spectrometer, Philips X'pert, Eindhoven, Netherlands).

#### ATR-FTIR analysis

The attenuated total reflection-Fourier transform infrared (ATR-FTIR) spectroscopy was used to characterize the functional groups of ZSM-5 particles, PEBA and PEBA containing 10 wt% ZSM-5 membranes. The FTIR spectra were achieved by a Nicolet Nexus 670 spectrometer (Nicolet Instrument Co., Madison, WI, USA) over a wave number range of 3200–650 cm^−1^.

#### FESEM analysis

The morphological characteristics and particle size of the synthesized ZSM-5 zeolite were investigated using field emission scanning electron microscopy (FESEM) analysis by a TESCAN microscope (VEGA 3SB, TESCAN, Czech Republic).

#### SEM analysis

The SEM images were carried out using a scanning electron microscopy (SEM) device (model S-4160, Hitachi, NJ, USA) after gold coating to observe the surface and cross-section morphologies of the PEBA/PES and ZSM-5/PEBA/PES membranes. For the cross-sectional SEM images, the samples were prepared by fracturing the water-wetted membrane in liquid nitrogen.

#### DSC analysis

The differential scanning calorimeter (DSC) analysis (Mettler-Toledo Inc., Switzerland) was carried out to determine the glass transient temperature (*T*_g_) of the neat PEBA and ZSM-5/PEBA membranes. The samples were heated under pure argon atmosphere at a heating rate of 10 °C min^−1^ from −100 to 220 °C.

#### Contact angle

The optical contact angle device (OCA-20, Data physics GmbH, Filderstadt, Germany) was used to determine the water contact angle and hydrophobicity of the fabricated membranes. The water contact angle was measured at room temperature on the surface of each membrane in at least three different points and the mean values were reported.

### Pervaporation experiments

To investigate the separation performance of the fabricated membrane, the pervaporation experiments were carried out using a cross flow flat plate and frame membrane module with an effective area of 35 cm^2^ for the separation of low concentrations of EAc from aqueous solutions. The membrane module had a feed channel with dimensions of 5 cm (width) × 7 cm (length) × 0.213 cm (height). The pervaporation apparatus was similar as described by Aroujalian and Raisi.^41^ Each experiment was repeated three times and the average values were reported. The performance of the prepared MMMs with different ZSM-5 zeolite concentrations (0, 5, 7.5, 10, 12.5 and 17.5 wt%) was investigated in a laminar feed flow at a feed concentration of 5 wt% and a temperature of 40 °C. Also, the effects of EAc concentration (1, 3 and 5 wt%) and operating temperature (30, 40 and 50 °C) at laminar (feed flow rate of 4 L min^−1^) and turbulent flow regimes (feed flow rate of 13 L min^−1^) were evaluated on the separation of EAc/water mixtures using the M0 and M10.0 membranes. The EAc concentration in the permeate solutions was specified using a gas chromatography (Younglin 6000 M Series Gas Chromatography, Anyang, Korea) equipped with a flame ionization detector (FID) and a TRB-Wax capillary column (Teknokroma, Barcelona, Spain) 60 m × 0.32 mm ID × 0.5 μm film thickness. The carrier gas was helium with a column pressure of 10 psi. The temperature of the oven, injector and detector were 100, 200 and 220 °C, respectively.

The performances of the membranes for pervaporation separation of EAc/water mixture can be indicated in terms of total permeate flux (*J*) and separation factor (*α*) as follows:1
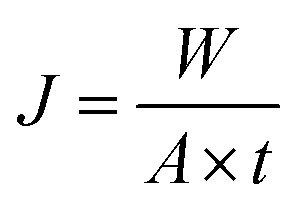
2
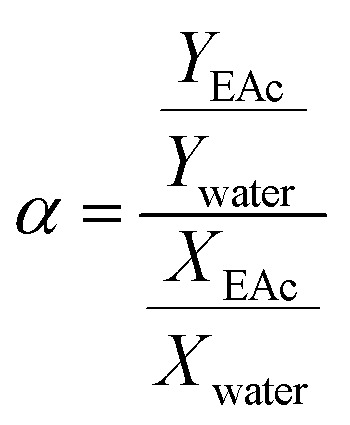
where *W*, *A*, *t*, *Y* and *X* are the mass of the collected permeate sample, the effective area of the membrane, process time and mass fractions of components in the permeate and feed solutions, respectively.

## Results and discussion

### ZSM-5 zeolite characterization

**Fig. 2:**
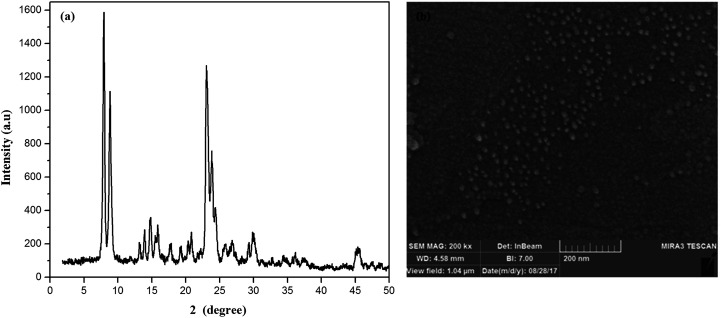
The XRD pattern (a) and FESEM image (b) of the synthesized ZSM-5 zeolite nanoparticles.

Fig. 2a shows the XRD pattern of the synthesized ZSM-5 zeolite. There are four sharp diffraction peaks at 2*θ* = 7.9, 8.8, 23.9 and 24.4° which are attributed to the ZSM-5 zeolite.^42^ The sharp peaks in the XRD pattern of zeolite indicated the pure crystalline structure of synthesized zeolites. The crystal size of zeolite particles obtained from the well-known Scherrer's equation was found to be 32.3 nm.

The FESEM image of ZSM-5 zeolite (Fig. 2b) showed that the almost spherical-like ZSM-5 particles with a uniform size distribution in the range of 20–50 nm were synthesized. The average particle size of ZSM-5 zeolite particles was 35 nm. The XRF result on the synthesized ZSM-5 zeolite indicated that the SiO_2_/Al_2_O_3_ ratio was found to be 52.

### Mixed matrix membranes characterization

#### Surface chemistry of membranes

**Fig. 3:**
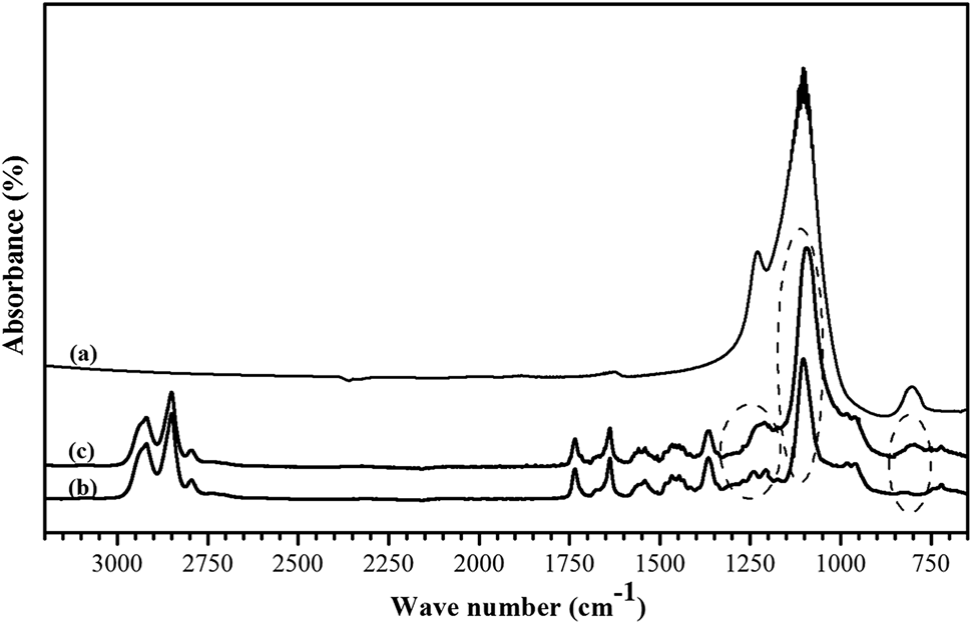
The ATR-FTIR spectra of the ZSM-5 zeolite (a), neat PEBA membrane (b) and PEBA membrane containing 10 wt% ZSM-5 (c).

The surface chemistry of the prepared membranes was characterized by the ATR-FTIR and water contact tests. The ATR-FTIR analysis was applied to detect the changes in the surface functional groups of the membrane in the presence of ZSM-5 particles. The ATR-FTIR spectra of the synthesized ZSM-5 zeolite, neat PEBA membrane and the PEBA membrane containing 10 wt% ZSM-5 are illustrated in Fig. 3. In ATR-FTIR spectrum of the ZSM-5 zeolite, the absorption bands at a wave number of 800 cm^−1^ is assigned to the SiO_4_ tetrahedron units and the strong absorption band in the range of 1000–1200 cm^−1^ is attributed to the vibration of SiO_4_ and AlO_4_ tetrahedra for the ZSM-5 zeolite.^43^ For both neat and mixed matrix membranes, the asymmetric CH_2_ bands are observed in the range of 2800–2900 cm^−1^. The stretching C=O at 1730 cm^−1^ and amide I adsorption band at 1640 cm^−1^ are assigned to urethane groups of PEBA. The peaks at wave numbers of 1350–1400, 1150–1240 and 720 cm^−1^ are related to CCH bending, amide III stretching group and stretching C–C group, respectively. The adsorption bands at a wave number of about 1100 and 800 cm^−1^ confirmed the ether function group in the PEO block of PEBA. The observed new bands at a wave number of 1100 and 800 cm^−1^ in the FTIR spectrum of MMM show the presence of ZSM-5 zeolite in the PEBA matrix.^40^

#### Contact angle

To investigate the hydrophobicity of the prepared pure PEBA and zeolite filled PEBA/PES membranes, the water contact angle measurements on the membranes were carried out. The results of contact angle on the neat PEBA/PES membrane and MMMs containing 5, 7.5, 10, 12.5 and 17.5 wt% ZSM-5 zeolite are given in Table 2. As shown, the contact angle of the prepared membranes was increased from 81 to 96° by increasing zeolite content in the PEBA/PES membranes from 0 to 17.5 wt% zeolite. These results proved enhancement in the hydrophobicity of MMMs by increasing the loading of the ZSM-5 zeolite nanoparticles content into the PEBA membrane. Similar observations were reported in previous studies. For example, Zhang *et al.*^26^ showed that the hydrophobicity of HTPB–PU/ZSM-5 membranes was enhanced from 88.5 to 93° with increasing ZSM-5 loading content from 0 to 20 wt%. Also, Gu *et al.*^30^ observed the enhancement in contact angle of zeolite filled PEBA membranes from 82 to 97° by increasing the zeolite concentration up to 40 wt%.

**Table 2:** The water contact angle of various membranes

Membrane	Contact angle (°)
M0	81 ± 2.1
M05.0	86 ± 1.9
M07.5	90 ± 2.9
M10.0	92 ± 1.7
M12.5	94 ± 2.5
M17.5	96 ± 1.6

#### Morphology of membranes

**Fig. 4:**
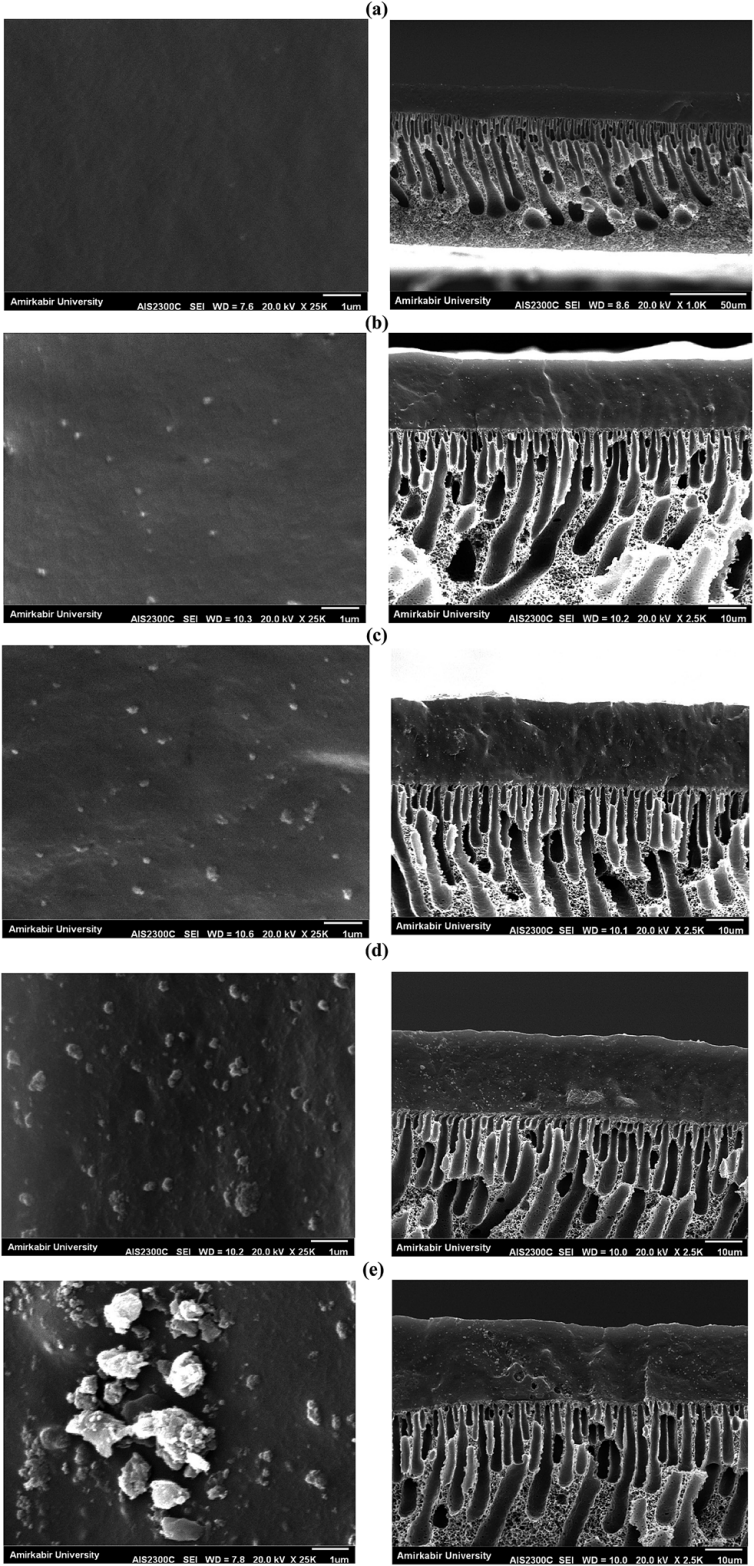
The SEM images of top surface (left) and cross section (right) of the prepared membranes: M0 (a), M05.0 (b), M10.0 (c), M12.5 (d) and M17.5 (e).

The SEM images of the top surface and cross-section of the PEBA/PES membrane and MMMs containing 5, 10, 12.5 and 17.5 wt% zeolite are illustrated in Fig. 4. As shown in Fig. 4a, the surface of the M0 membrane was smooth without any pores. The surface images of MMMs indicated that the bright spots on the membrane surface were increased by increasing zeolite concentration which represents the zeolite particles on the top surface of the MMMs. Obviously, the zeolite content on the membrane surface increased by enhancement of incorporated zeolites into the membranes. The ZSM-5 particles were well dispersed in the MMMs containing 5 and 10 wt% zeolite. The lower zeolite loading in MMMs resulted in good distribution of zeolite particles on the membrane surface. This is while, the zeolite particles were aggregated in ZSM-5/PEBA/PES MMMs containing 12.5 and 17.5 wt% zeolite. The agglomeration of zeolite particles on the membrane surface containing 17.5 wt% zeolite was higher than that of the MMMs surface containing 12.5 wt% zeolite. The cross-sectional SEM image of the neat PEBA/PES membrane indicated that the prepared PEBA/PES defect-free has a dense top PEBA layer with a thickness of about 20 ± 2 μm on a porous PES support with a finger like structure and thickness of about 80 ± 5 μm. The cross-section SEM images of the zeolite filled PEBA/PES membranes showed that the ZSM-5 zeolite particles were distributed on the top layer of MMMs. As shown in Fig. 4b and c, the zeolite particles were uniformly dispersed in the PEBA network without any agglomeration and void in the zeolite/polymer interface of MMMs containing 5 and 10 wt% zeolite. This is while, the agglomeration of the zeolite particles is observed in the cross-sectional SEM images of ZSM-5/PEBA/PES MMM containing 12.5 and 17.5 wt% zeolite.

#### Thermal behavior of membranes

**Fig. 5:**
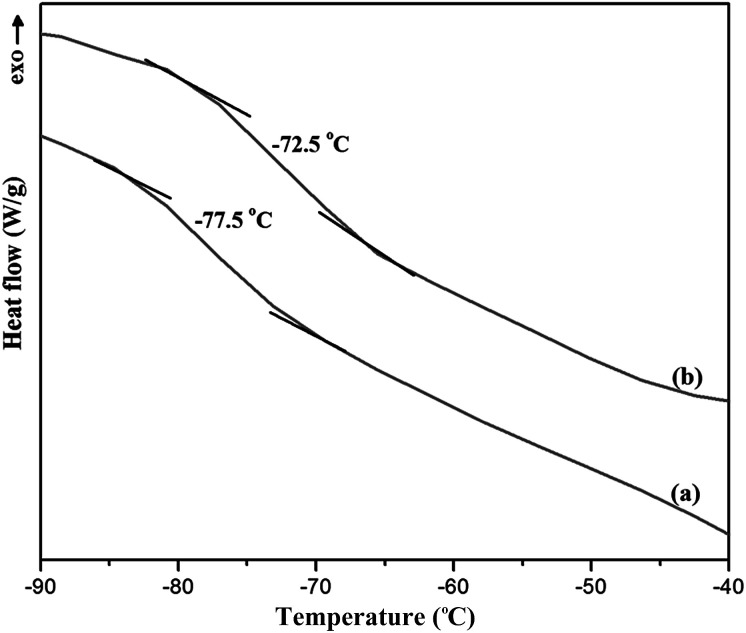
The DSC curves of the neat PEBA membrane (a) and PEBA membrane containing 10 wt% ZSM-5 (b).

The DSC analysis was used to determine the thermal decomposition behavior of the prepared membranes and the interaction between the PEBA and ZSM-5 zeolite. Typically, the DSC plots of the neat PEBA and PEBA containing 10 wt% ZSM-5 membranes are illustrated in Fig. 5. As shown, by adding ZSM-5 zeolite into the polymer matrix, the glass transient temperature (*T*_g_) was increased from −77.5 °C for the neat PEBA membrane to −72.5 °C for the ZSM-5 filled PEBA membrane containing 10 wt% zeolite. The increasing *T*_g_ could be attributed to rigidifying of the polymer chains around the zeolite particles and increasing interfacial interaction. Zarshenas *et al.*^40^ indicated that the *T*_g_ of the pure Pebax-1657 membrane increased from −57.83 °C to −50.42 °C by loading 4 wt% NaX nanozeolite into the PEBA membrane. Yu *et al.*^44^ also observed the enhancement in *T*_g_ of the Pebax-1657 membrane by loading different fillers including silica nanoparticles, polystyrene (PS) colloids and carbon nanotubes (CNTs) into the PEBA membrane. The *T*_g_ of −77.1 for pure PEBA-2533 was reported by Rahman *et al.*^45^

### Mixed matrix membranes pervaporation performance

#### Effect of zeolite loading concentration

**Fig. 6:**
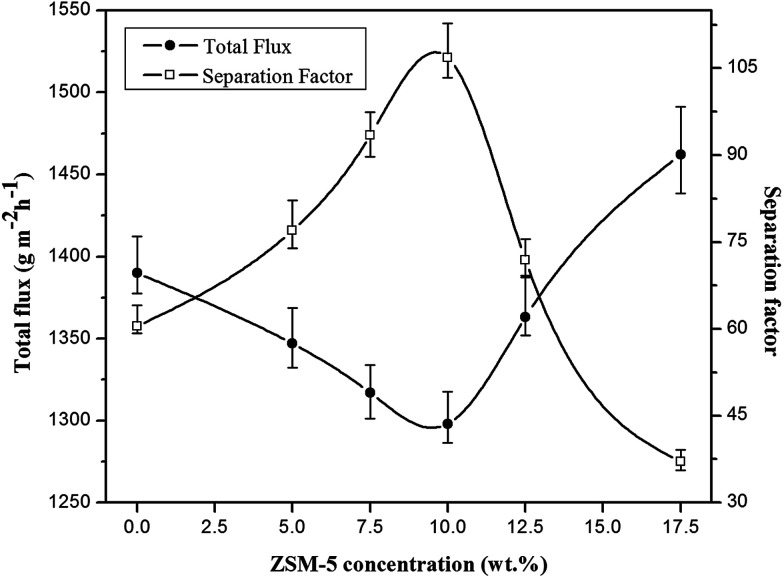
The effect of ZSM-5 concentration on the total flux and separation factor of various prepared membranes (EAc concentration = 5 wt% and *T* = 40 °C).

The effect of zeolite loading content in the ZSM-5 filled PEBA/PES dual-layer membranes on the pervaporation performance for a feed concentration of 5 wt% EAc at operating temperature of 40 °C in the laminar flow regime is illustrated in Fig. 6. As indicated in this figure, the separation factor was significantly increased by an enhancement in the zeolite content in membranes up to 10 wt% and a further increase in the zeolite concentration resulted in a decrease in the separation factor. The gradual decrease in the total permeate flux was obtained by increasing ZSM-5 loading up to 10 wt% zeolite. By loading zeolite into the PEBA/PES membranes, the EAc and water molecular movement were decreased in the membrane matrix and consequently, the decrease in total permeation flux of the zeolite filled PEBA/PES membranes was observed compared with the neat PEBA/PES dual-layer membrane. The decrease in the EAc and water movement in the membrane matrix after loading ZSM-5 can be attributed to the polymer chain rigidification after incorporation of ZSM-5 nanoparticles into the polymer matrix. In the mixed matrix membranes, the mobility of the polymer chains in the region directly contacting the inorganic particles can be inhibited relative to that for the bulk polymer, this phenomenon is called polymer chain rigidification. The polymer chain rigidification can be easily detected by increasing *T*_g_ with the addition of zeolite nanoparticles.^46^ The inhibited movement of the polymer chains near the zeolite particle is the major cause of *T*_g_ shift.^47^ As confirmed by the DSC analysis, the rigidification of polymer chains was observed through the increasing of *T*_g_ by loading the zeolite into the PEBA/PES membrane.

The permeation flux was increased at zeolite concentrations higher than 10 wt%. Defects in the top layer of the MMMs could be responsible for increasing the permeation flux of the dual-layer membrane containing 12.5 and 17.5 wt% ZSM-5. These defects were created in the membrane top layer due to agglomeration of ZSM-5 nanoparticles at high zeolite concentrations, as detected by the SEM analysis (Fig. 4d and e). The increase in separation factor by increasing zeolite loading content up to 10 wt% could be attributed to the enhancement in hydrophobicity of the membrane and increasing the sorption of EAc molecules by adding ZSM-5 zeolite into the PEBA/PES membranes as confirmed by contact angle tests. Based on the results of the contact angle test, by loading ZSM-5 zeolite into the PEBA matrix, the contact angle of the prepared membranes was increased which resulted in more selective sorption of the EAc molecules using the MMMs. The aggregation of ZSM-5 nanoparticles in MMMs at concentrations higher than 10 wt% zeolite resulted in defects between the polymer chains, which allowed the simultaneous permeation of EAc and water and led to a decrease in the separation factor. Therefore, the best separation performance was observed for the dual-layer ZSM-5/PEBA/PES membrane containing 10 wt% zeolite. Zhang *et al.*^26^ indicated that the optimum performance of the HTPB–PU/ZSM-5 membrane was obtained by loading 20 wt% ZSM-5 into the membranes for pervaporation separation of isopropyl acetate from aqueous solution. Gu *et al.*^30^ observed the enhancement in EAc/water separation factor by enhancement of the ZSM-5 loading from 0 to 10 wt% into the single layer PEBA membrane.

#### Effect of pervaporation operating parameters

**Fig. 7:**
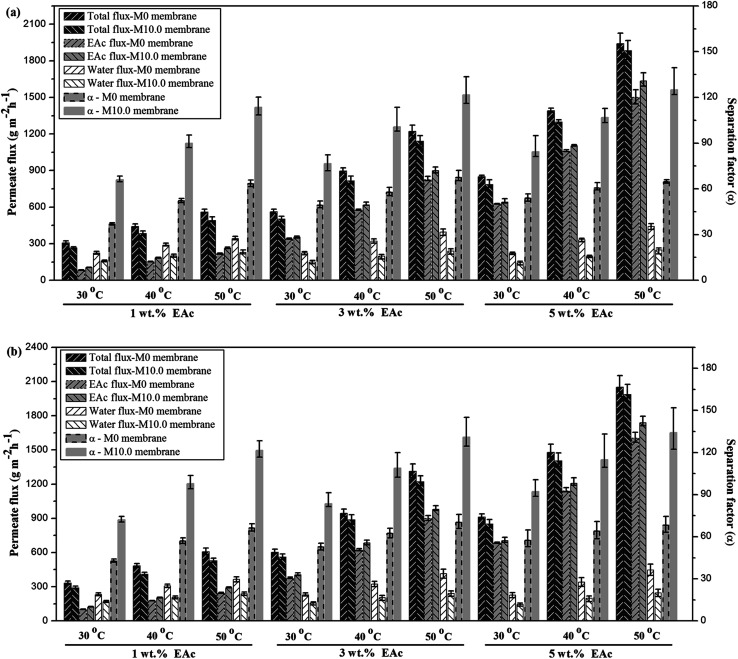
The effect of feed concentration and temperature on the total, EAc and water fluxes and also separation factor for the M0 and M10.0 membranes at laminar (a) and turbulent (b) feed flow regimes.

The simultaneous effects of feed concentration, temperature and feed flow regime on the separation factor, water and EAc partial fluxes and total flux of the M0 and M10.0 membranes are illustrated in Fig. 7. In the following, a parametric study was done to investigate the influence of pervaporation operating parameters on the separation performance of the prepared membranes.

**Fig. 8:**
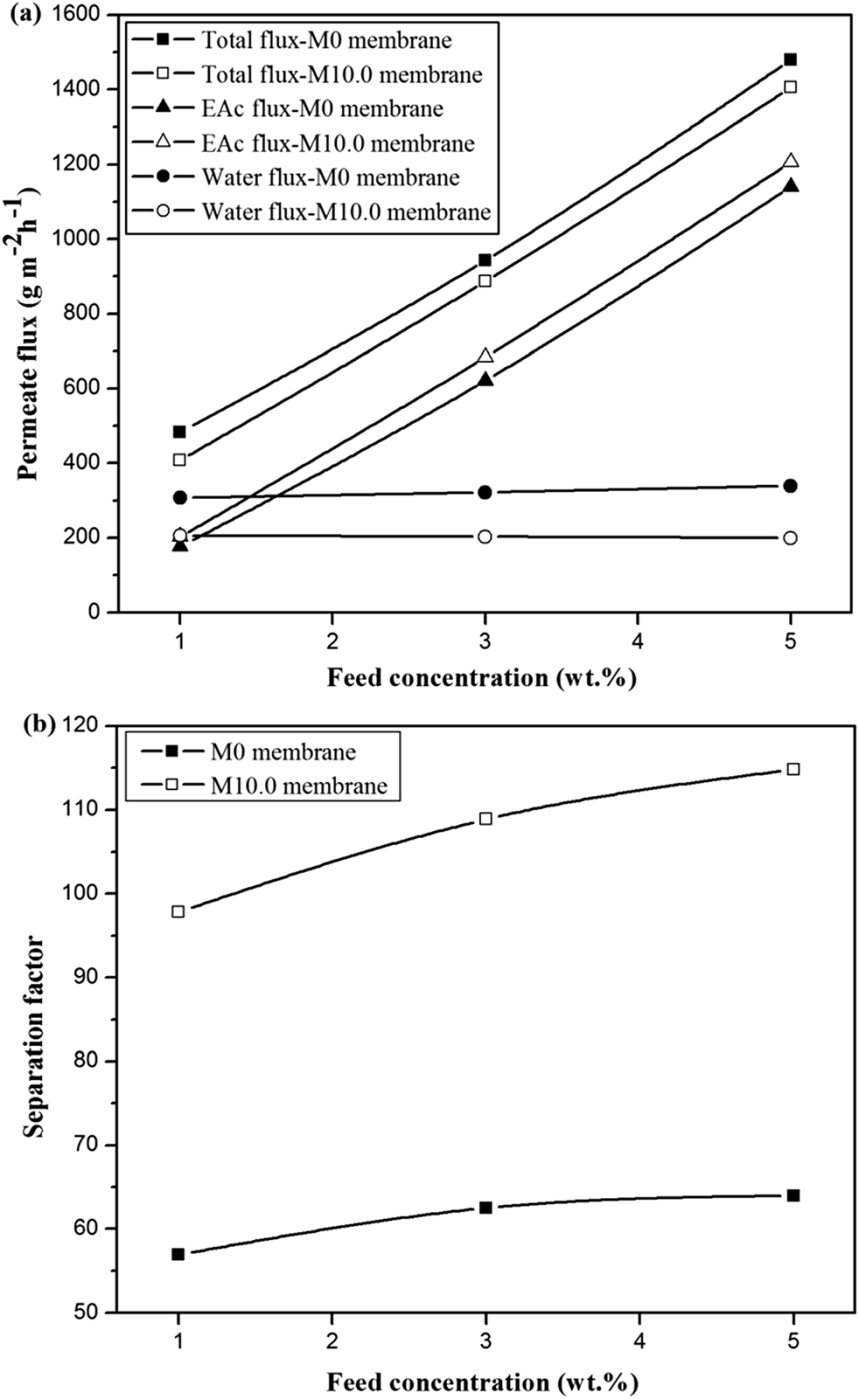
The effect of feed concentration on the permeate fluxes (a) and separation factor (b) for the M0 and M10.0 membranes at temperature of 40 °C and turbulent flow.

The comparison of the separation factor of both M0 and M10.0 membranes at the turbulent flow regime showed that the separation factor of both membranes in the turbulent flow regime was higher than the separation factor at laminar flow regime in different feed concentrations and operating temperatures as indicated in Fig. 7. The EAc permeation flux and total flux for both M0 and M10.0 membranes at the turbulent flow regime were higher than the laminar flow regime. By increasing the feed flow rate, the mass transfer rate of EAc from the bulk solution to the membrane surface was increased and subsequently the EAc partial flux and total flux were enhanced. This trend can be explained based on the resistance-in-series model in which the total mass transport resistance through a dual-layer membrane is the sum of the liquid boundary-layer resistance, intrinsic membrane active layer resistance and support layer resistance.^48^ When the liquid boundary-layer resistance is much larger than the intrinsic membrane resistance, the mass transfer in the boundary-layer becomes rate-limiting.^49^ Therefore, a change in the feed flow regime from laminar to turbulent leads to a decrease in the concentration polarization near the membrane surface and a reduction in the boundary-layer thickness, and consequently the permeation rate across the membrane enhances. These observations are consistent with the results in the literature. For instance, Dutta and Sikdar^50^ reported enhancement in the separation factor of trichloroethane with increasing feed flow rate. Psaume *et al.*^51^ indicated that the permeation flux was increased by increasing the Reynolds number. Li *et al.*^52^ observed an enhancement in both total flux and separation factor of the cellulose acetate/polydimethylsiloxane membrane for the pervaporation separation of acetone from aqueous solution by increasing flow rate. The permeation fluxes and separation factor as a function of feed concentration for the M0 and M10.0 membranes at a temperature of 40 °C and turbulent feed flow is indicated in Fig. 8. As shown, the separation factor, total flux and EAc partial flux of both membranes increased by variation in feed concentration from 1 to 5 wt%. The composition of feed solution has a remarkable influence on the component sorption into the membrane and on the component diffusion through the membrane, because the kinetic and thermodynamic properties of the permeating species and membrane are significantly affected by the component concentration in the feed solution. The enhancement in EAc permeation flux by increasing feed concentration could be attributed to increasing the driving force and acceleration of the diffusion rate of EAc molecular during the pervaporation process. Increasing EAc feed concentration will enhance the concentration gradient across the membrane; thereby the EAc permeation rate through the membrane increases. Also, the high sorption of EAc relative to water into the membranes due to the hydrophobic nature of the membrane is responsible for increasing the separation factor by increasing the EAc concentration in the feed solution. As confirmed by contact angle and FTIR tests, the hydrophobic structure of the PEBA/PES and ZSM-5/PEBA/PES membranes resulted in the faster sorption rate of EAc compared with the sorption rate of water and an enhancement in interaction between the EAc and membrane resulted in an increase in the separation factor by an enhancement in EAc concentration. The gradual increase in the water partial flux was observed by increasing EAc feed concentration for the M0 membrane, whereas the insignificant change in water flux was achieved by increasing feed concentration for the M10.0 membrane, as indicated in Fig. 8a. This behavior can be ascribed to the membrane swelling phenomenon. The membrane swelling due to an increase in the feed concentration affects the separation performance of the membrane. The swelling of the membrane leads to a membrane with higher free volume and unrestricted transfer of the permeating components through the membrane. When a higher EAc concentration feed solution was in contact with the neat PEBA/PES membrane, the membrane swelling occurred. This resulted in easier transport of water molecules through the swollen membrane, and consequently the water partial flux enhanced with the EAc feed concentration. The constant water permeation flux with an enhancement in the feed concentration for the M10.0 membranes indicated that the incorporation of ZSM-5 nanoparticles into the PEBA matrix prevents the membrane swelling.

**Fig. 9:**
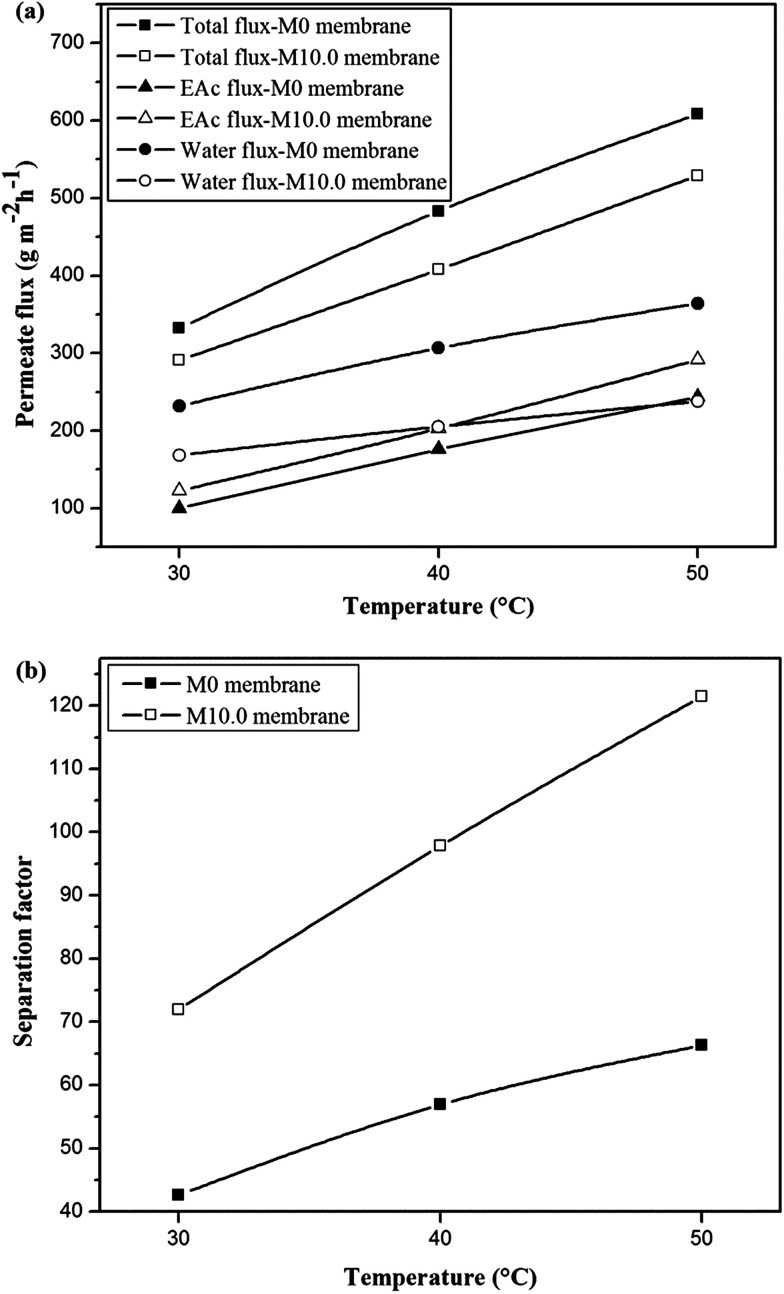
The effect of feed temperature on the permeation fluxes (a) and separation factor (b) for the M0 and M10.0 membranes for EAc feed concentration of 1 wt% at turbulent flow.

The permeation fluxes and separation factor as a function of feed temperature for the M0 and M10.0 membranes for the EAc feed concentration of 1 wt% and turbulent feed flow is shown in Fig. 9. It can be seen that all permeation fluxes and EAc separation factor increased by enhancing the feed temperature. Since the viscosity and diffusivity of components in the feed solution as well as the solubility and diffusivity of permeating components in the membrane are temperature dependent, a change in the operating temperature has a significant influence on the separation properties of the membrane. Moreover, in the dense polymeric membranes, the permeating components diffuse in the membrane *via* the free volumes, which are produced by jumping of the polymer chains. The frequency of polymer chain motions enhances by increasing temperature. Consequently the free volume in the polymer matrix increases and leads to higher permeation rate through the membrane. Therefore, both EAc and water partial fluxes and total flux enhance as the operating temperature goes to higher levels. Furthermore, Fig. 9b indicates that the separation factor enhanced by increasing the operating temperature. The EAc and water partial fluxes enhance with temperature, but the EAc flux is more sensitive to variation in the feed temperature because of its higher activation energy for the permeation. Thus, the separation factor that is proportional to the ratio of the EAc flux to water flux increases by enhancing the operating temperature. Similar reports are presented in the literature for the effect of feed concentration and temperature on the permeation flux and separation factor. For example, Zhang *et al.*^26^ observed an enhancement in the isopropyl acetate and water partial flux, total flux and separation factor using the HTPB–PU/ZSM-5 membrane by increasing feed concentration from 0.2 to 1 wt% and operating temperature from 30 to 60 °C. Gu *et al.*^30^ investigated the effect of feed concentration and temperature on the pervaporation performance of the ZSM-5/PEBA membrane toward low concentrations of EAc from aqueous solution. They found the significant enhancement in the EAc partial flux, total flux and separation factor as well as gradual reduction in the water partial flux by increasing feed concentration. Both separation factor and total flux were also enhanced by increasing the feed temperature up to 50 °C. Tan *et al.*^53^ also observed that both separation factor and total permeation flux for separation of *n*-butanol/water mixtures using the ZSM-5/PEBA membrane were increased by enhancing temperature and feed concentration.

#### Statistical analysis

**Fig. 10:**
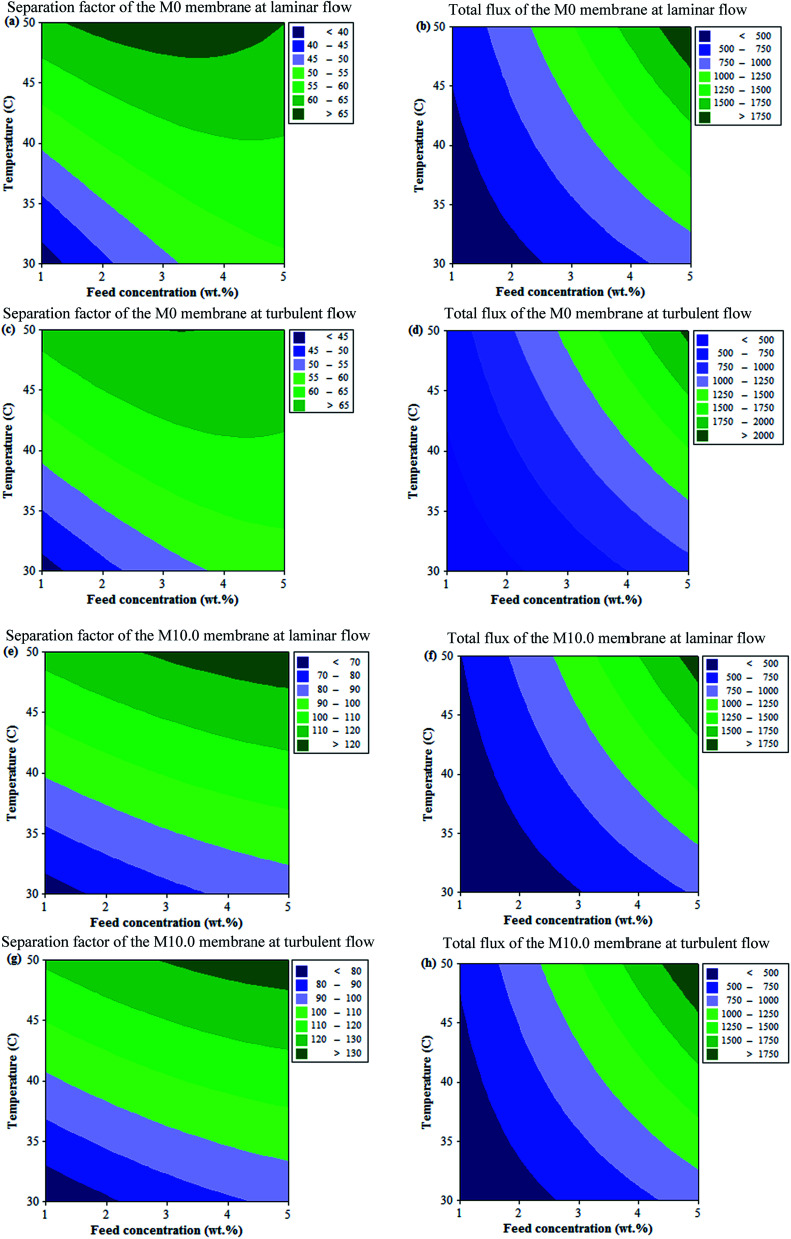
The counter plots of the M0 and M10.0 membranes.

Analysis of variance (ANOVA) was used to investigate the interaction effect of feed concentration and operating temperature on the separation factor and total permeation flux of the M0 and M10.0 membranes as well as the simultaneous optimization of parameters for the optimum performance of the membranes at laminar and turbulent flow regimes. The ANOVA results for the separation factor and total permeation flux of the M0 and M10.0 membranes as a function of feed concentration and operating temperature are listed in Tables 3 and 4, respectively. The large value of *F* indicates that most of the variations in the response could be explained by the regression equation. The *P*-value lower than 0.05, indicates the significant term in surface response analysis. By elimination of insignificant terms (*p* > 0.05) from the full quadratic model, the polynomial equations for the separation factor and total permeation flux of the membranes can be expressed as follows:

3 *Y* = *β*_0_ + *β*_1_*X*_1_ + *β*_2_*X*_2_ + *β*_3_*X*_1_^2^ + *β*_4_*X*_2_^2^ + *β*_5_*X*_1_*X*_2_

where *Y* is the predicted response by the model, *X*_1_ and *X*_2_ are the feed concentration and operating temperature and *β*_0_ to *β*_5_ are the constant regression coefficient of the model. All parameters of eqn (3) are reported in Table 5. As shown in Table 5, both variables, *i.e.* feed concentration and temperature, had a positive effect on the separation factor and total flux of the prepared membranes at laminar and turbulent flow regimes. Moreover, the temperature had a higher effect on the separation factor compared with the feed concentration, as the *F* value of temperature was higher than that of the EAc feed concentration. Based on the *F* values (Tables 3 and 4), the singular effect of each variable was higher than the interactional effect of feed concentration and temperature. The counter plots for the measured responses as a function of feed concentration and operating temperature are illustrated in Fig. 10. The obtained results revealed that the simultaneous enhancement in the feed concentration and operating temperature resulted in increasing the separation factor and total flux of the prepared membranes. Also, the separation factor and total flux of both membranes at the turbulent flow regime were higher than the separation factor and total flux at laminar flow regime in the same condition of feed concentration and temperature. For example, by solving the statistical models and optimization of variables for 1 wt% EAc at a temperature of 30 °C, the separation factor and total flux of the M0 membrane were estimated to be 37.57 and 312.73 g m^−2^ h^−1^, respectively at the laminar flow regime and 43.15 and 335.7 g m^−2^ h^−1^ at the turbulent flow regime, respectively. For the M10.0 membrane at the same condition, the separation factor and total flux were estimated to be 65.88 and 270.98 g m^−2^ h^−1^ at the laminar flow regime, respectively and 72.10 and 294.43 g m^−2^ h^−1^ at turbulent flow regime respectively. The predicted values for the separation factor and total flux of membranes by models were in good agreement with the obtained experimental values.

**Table 3:** The ANOVA results for the M0 membranes

Source	DF	Laminar flow regime						Turbulent flow regime					
		Separation factor			Total flux			Separation factor			Total flux		
		Sum of squares	*F* factor	*P* factor	Sum of squares	*F* factor	*P* factor	Sum of squares	*F* factor	*P* factor	Sum of squares	*F* factor	*P* factor
Regression	5	713.367	154.60	0.001	2 217 883	10 779.99	0.000	604.314	417.71	0.000	2 458 380	5845.55	0.000
Linear	2	633.376	343.16	0.000	2 041 397	24 805.46	0.000	544.613	941.10	0.000	2 271 367	13 502.18	0.000
*X*_1_	1	119.260	129.23	0.001	1 374 731	33 409.30	0.000	92.905	321.08	0.000	1 518 054	18 048.2	0.000
*X*_2_	1	514.115	557.09	0.000	666 667	16 201.62	0.000	451.707	1561.12	0.000	753 313	8956.16	0.000
Square	2	19.151	10.38	0.045	924	11.23	0.040	18.741	32.39	0.009	1252	7.44	0.069
*X*_1_ × *X*_1_	1	17.543	19.01	0.022	910	22.12	0.018	13.957	48.24	0.006	1250	14.86	0.031
*X*_2_ × *X*_2_	1	1.608	1.74	0.279	14	0.35	0.598	4.784	16.53	0.027	2	0.02	0.887
Interaction	1	60.840	65.93	0.004	175 561	4266.56	0.000	40.960	141.56	0.001	185 761	2208.52	0.000
*X*_1_ × *X*_2_	1	60.840	65.93	0.004	175 561	4266.56	0.000	40.960	141.56	0.001	185 761	2208.52	0.000
Residual error	3	2.769			123			0.868			252		
Total	8	716.135			2 218 006			605.182			2 458 632		

**Table 4:** The ANOVA results for the M10.0 membranes

Source	DF	Laminar flow regime						Turbulent flow regime					
		Separation factor			Total flux			Separation factor			Total flux		
		Sum of squares	*F* factor	*P* factor	Sum of squares	*F* factor	*P* factor	Sum of squares	*F* factor	*P* factor	Sum of squares	*F* factor	*P* factor
Regression	5	3344.74	1110.54	0.000	2 163 062	1621.45	0.000	3664.84	2246.74	0.000	2 403 742	5552.44	0.000
Linear	2	3321.08	2756.71	0.000	1 971 024	3693.75	0.000	3635.56	5571.96	0.000	2 201 550	12 713.47	0.000
*X*_1_	1	362.70	602.14	0.000	1 330 104	4985.29	0.000	414.00	1269.03	0.000	1 512 024	17 463.22	0.000
*X*_2_	1	2958.37	4911.29	0.000	640 920	2402.20	0.000	3221.56	9874.90	0.000	689 526	7963.73	0.000
Square	2	12.21	10.13	0.046	2377	4.45	0.126	15.59	23.90	0.014	1040	6.01	0.089
*X*_1_ × *X*_1_	1	8.50	14.11	0.033	2112	7.92	0.067	11.46	35.12	0.010	968	11.18	0.044
*X*_2_ × *X*_2_	1	3.71	16.16	0.049	264	0.99	0.393	4.14	12.68	0.038	72	0.83	0.429
Interaction	1	11.46	19.02	0.022	189 660	710.86	0.000	13.69	41.96	0.007	201 152	2323.22	0.000
*X*_1_ × *X*_2_	1	11.46	19.02	0.022	189 660	710.86	0.000	13.69	41.96	0.007	201 152	2323.22	0.000
Residual error	3	1.81			800			0.98			260		
Total	8	3346.55			2 163 862			3665.82			2 404 002		

**Table 5:** The predicted response and constant parameters of eqn (3)

*Y*	*β*_0_	*β*_1_	*β*_2_	*β*_3_	*β*_4_	*β*_5_
Separation factor of the M0 membrane at laminar flow	−15.61	14.47	1.51	−0.74	0.000	−0.195
Total flux of the M0 membrane at laminar flow	147.00	−211.00	1.91	5.33	0.000	10.47
Separation factor of the M0 membrane at turbulent flow	−27.62	12.33	2.58	−0.66	−0.015	−0.16
Total flux of the M0 membrane at turbulent flow	129.75	−217.00	3.11	6.25	0.000	10.78
Separation factor of the M10.0 membrane at laminar flow	−36.52	10.36	3.56	−0.51	−0.013	−0.085
Total flux of the M10.0 membrane at laminar flow	184.38	−248.83	0.02	8.13	0.000	10.89
Separation factor of the M10.0 membrane at turbulent flow	−35.89	11.44	3.75	−0.59	−0.014	−0.092
Total flux of the M10.0 membrane at turbulent flow	175.33	−230.50	0.26	5.50	0.000	11.21

### Comparison of pervaporation performance

The pervaporation separation of EAc from aqueous solution using the prepared membranes at optimum conditions was compared with other PEBA and MMMs reported by other researchers and the results are listed in Table 6. As shown, the total flux of the prepared MMMs in this work is higher than those of other reported membranes in the literature. The good balance in the trade-off between the permeation flux and the separation factor of the prepared membrane was observed. The use of ZSM-5 zeolite increased the EAc separation factor of the M10.0 membrane.

**Table 6:** The pervaporation performance of membranes for separation of ethyl acetate from aqueous solution

Membrane	Feed (wt%)	*T* (°C)	Flux (g m^−2^ h^−1^)	Separation factor	Ref.
PEBA/ZSM-5	5	50	199.5	185	30
P(VDF-HFP)/[bmim]BF4	5	45	737	123	54
P(VDF-HFP)	5	45	510	65	54
PDMS/PTFE	0.99	30	276	95	55
P(VDF-*co*-HFP)	3	30	690	163	56
P(VDF-HFP)	3	30	415	80	57
PDMS	5	40	460	76	58
PDMS/PMHS	5	40	260	24	58
Polyurethane urea	2.5	30	250	655	59
M0	5	50	1940	64.70	This study
M10.0	5	50	1985	134.22	

## Conclusion

The EAc was successfully separated from its aqueous solution by the pervaporation process with the prepared ZSM-5/PEBA/PES dual-layer mixed matrix membranes. The influences of zeolite content of the membrane and operating parameters of the pervaporation process like feed concentration, temperature and feed flow regime on the separation performance of the prepared membranes were investigated. The ZSM-5 zeolite nanoparticles were synthesized *via* the conventional hydrothermal method. The XRD pattern of ZSM-5 zeolite showed the crystalline structure of the synthesized zeolite particles. The SEM images of the prepared membranes indicated the homogenous dispersion of ZSM-5 zeolite in the top layer of the membranes up to 10 wt% zeolite, while the aggregation of ZSM-5 nanoparticles in the top layer was observed by zeolite loading concentrations higher than 10 wt%. The contact angle measurement indicated that the hydrophobicity and glass transient temperature of membranes was increased by loading ZSM-5 zeolite into the PEBA/PES dual-layer membranes. The maximum separation factor of 106.83 and total flux of 1298 g m^−2^ h^−1^ were obtained by loading 10 wt% ZSM-5 zeolite into the PEBA matrix for feed concentration of 5 wt% and temperature of 40 °C at laminar feed flow. The results of pervaporation experiments showed that the separation factor, total permeation flux and EAc partial flux of both neat and mixed matrix membranes were increased by increasing feed concentration from 1 to 5 wt% and temperature from 30 to 50 °C. The water partial flux of MMMs did not change by increasing feed concentration, whereas an enhancement in the operating temperature resulted in a gradual increase in the water flux. The comparison of the membranes performance for the separation of EAc from EAc/water mixtures in the laminar and turbulent feed flow regimes revealed that the separation factor of both M0 and M10.0 membranes at the turbulent flow regime was higher than the separation factor values at the laminar flow regime. The EAc partial flux and total flux for both M0 and M10.0 membranes at the turbulent flow regime were higher than those of the laminar flow regime, whereas the water flux was almost constant at laminar and turbulent flow regimes for MMMs. The ANOVA results were evaluated for the total flux and separation factor of the M0 and M10.0 membranes as functions of feed concentration and operating temperature at laminar and turbulent flow. It was found that the temperature had a higher effect on the separation factor compared with the feed concentration, as the *F* value of temperature was higher than that of the EAc feed concentration.

## Conflicts of interest

There are no conflicts to declare.

## Supplementary Material
